# Associations between psychological needs satisfaction, humor styles, and psychological distress in emerging adults

**DOI:** 10.3389/fpsyg.2026.1793980

**Published:** 2026-04-13

**Authors:** Nataliia Maronchuk, Timo Schurr, Lorenz Michael Pammer, Fabienne Post, Alex Hofer

**Affiliations:** Division of Psychiatry I, Department of Psychiatry, Psychotherapy, Psychosomatics and Medical Psychology, Medical University Innsbruck, Innsbruck, Austria

**Keywords:** basic psychological needs (BPN), coping, emerging adulthood, humor styles questionnaire (HSQ), motivational perspective, psychological distress, self-determination theory (SDT)

## Abstract

**Introduction:**

Emerging adulthood is a developmental period marked by increased psychological vulnerability due to rising demands related to autonomy, identity development, and social relationships. Self-determination theory (SDT) posits that satisfaction of the basic psychological needs for autonomy, competence, and relatedness is essential for psychological adjustment, whereas unmet needs are associated with psychological distress. While the direct association between needs satisfaction and distress is well documented, less is known about the psychological processes that may account for this relationship. Humor may represent one such process, as it is a common emotion-regulation and interpersonal coping strategy. However, humor styles differ in their adaptability, and potential sex differences in these pathways remain insufficiently explored.

**Methods:**

This cross-sectional study examined 226 university students aged 18–30 years (67.3% female). Participants completed self-report measures of psychological distress, psychological needs satisfaction, and humor styles. Correlation analyses, mediation models, and moderated mediation models were used to examine the associations between psychological needs satisfaction, humor styles, and psychological distress as well as sex-specific differences in these relationships. Higher satisfaction of psychological needs was associated with lower psychological distress, with relatedness showing the strongest association.

**Results:**

Higher levels of needs satisfaction were positively associated with adaptive humor styles, particularly self-enhancing humor, and negatively associated with self-defeating humor. Self-enhancing humor was negatively correlated with psychological distress, while self-defeating humor was positively correlated with it. Statistically, both humor styles accounted for part of the association between psychological needs satisfaction and distress. Moderated mediation analyses revealed sex-specific patterns: the indirect association between autonomy satisfaction and distress via self-defeating humor was stronger in males. In contrast, the indirect association via self-enhancing humor was stronger in females, particularly with regard to relatedness and competence.

**Discussion:**

These findings underscore the significance of humor styles as correlates of motivational processes and psychological distress in emerging adulthood. Psychological needs satisfaction is associated with lower distress, in part due to adaptive emotion-regulation tendencies reflected in humor use. Distinct patterns are observed for men and women. These results emphasize the importance of considering motivational context, coping tendencies, and sex differences when examining mental health during this critical developmental period.

## 1 Introduction

Emerging adulthood is a developmental period characterized by significant psychological and social changes. The period between the ages of 18 and 30 is often marked by increased instability due to mounting expectations related to autonomy, identity formation, academic or professional performance, and social relationships (Arnett, 2000; Arnett, 2007). Most mental health conditions first appear during this life stage (McGrath et al., 2023). These challenges, coupled with elevated vulnerability to psychological distress, including symptoms of stress, anxiety, and depression, make emerging adulthood a critical period for developing adaptive coping strategies and determining long-term mental health trajectories (Kessler et al., 2005; Kessler et al., 2007).

Self-determination theory (SDT) (Deci and Ryan, 2000; Ryan and Deci, 2000) is a well-established framework for understanding psychological adjustment during this life phase. According to SDT, psychological well-being depends on satisfying three basic psychological needs: relatedness (feeling connected and belonging), competence (feeling effective and masterful), and autonomy (feeling volitional and self-endorsing actions) (Neubauer and Voss, 2016). Empirical evidence indicates that satisfying these needs is associated with enhanced well-being, while unmet needs are linked to psychological distress and maladaptive functioning (Ryan and Deci, 2000; Vansteenkiste and Ryan, 2013). Among emerging adults, unmet psychological needs have been shown to predict higher levels of stress, emotional dysregulation, and internalizing symptoms. This underscores the importance of motivational processes in mental health during this developmental stage (Sheldon et al., 2001; Neubauer and Voss, 2016).

The direct link between psychological needs satisfaction and distress is well-documented. However, the behavioral and emotional processes through which motivational states lead to psychological outcomes are less understood. One such process may involve humor, a common and socially embedded coping strategy. Humor can serve as a means of regulating emotions, reappraising cognitions, and interacting with others, helping individuals to manage stress and negative emotions (Martin, 2007; Samson and Gross, 2012; Samson and Gross, 2014). However, humor is not a unidimensional concept. It consists of separate styles that vary in psychological adaptability.

The Humor Styles Model distinguishes four styles: affiliative (enhancing social relationships), self-enhancing (maintaining a positive outlook in adversity), aggressive (teasing or disparaging others), and self-defeating (self-disparagement to gain social approval) (Martin et al., 2003). Research shows that affiliative and self-enhancing humor are associated with better psychological adjustment. In contrast, self-defeating humor is linked to lower self-esteem, rumination, and higher psychological distress (Martin et al., 2003; Tucker et al., 2014). Aggressive humor, by contrast, demonstrates more context-dependent associations with mental health and interpersonal functioning (Cann et al., 2008; Martin and Ford, 2018).

From a motivational perspective, humor styles can be conceptualized as relatively stable tendencies for regulating emotions and coping with interpersonal situations, which are embedded in an individual’s broader psychological and social context (Martin et al., 2003; Samson and Gross, 2012). More specifically, the satisfaction or frustration of basic psychological needs has been linked to differences in emotion regulation and interpersonal functioning. These differences may be reflected in individuals’ habitual use of particular humor styles (Deci and Ryan, 2000; Martin, 2007; Ryan and Deci, 2017). In this sense, humor is not directly related to the satisfaction of needs. However, humor can be a behavioral pathway through which motivational states have psychological consequences (Dozois et al., 2009; Vansteenkiste and Ryan, 2013). Thus, examining humor as a mediating mechanism between the satisfaction of psychological needs and distress could provide insight into the processes linking motivation and mental health in emerging adulthood.

Furthermore, evidence suggests that these processes may differ by sex. Men and women exhibit systematic differences in emotion regulation, coping strategies, and humor usage, which likely reflect gender-related socialization patterns (Matud, 2004; Nolen-Hoeksema, 2012). Previous research indicates that women tend to use affiliative humor more frequently than men, who more often engage in aggressive or self-defeating humor (Martin et al., 2003; Cann et al., 2008). Sex differences have also been reported in the importance of specific psychological needs for well-being, particularly the need for autonomy (Saroglou and Scariot, 2002; Frewen et al., 2008). Together, these findings suggest that the relationships between psychological needs satisfaction, humor styles, and psychological distress may differ by sex. This highlights the need to examine moderated pathways.

The present study aimed to investigate the associations between psychological needs satisfaction, humor styles, and psychological distress in emerging adults to close this gap. Using a sample of university students, we examined the following: (i) the direct associations between psychological needs satisfaction, humor styles, and psychological distress; (ii) whether humor styles statistically account for the association between psychological needs satisfaction and distress; and (iii) whether these associations differ between men and women. Due to its cross-sectional design, the study did not seek to establish causal effects. Rather, it aimed to identify associative pathways that could inform future longitudinal and experimental research on motivation, coping, and mental health in emerging adulthood.

Based on previous findings, the following hypotheses were tested: (H1) Greater satisfaction of the psychological needs for autonomy, competence, and relatedness is associated with lower levels of psychological distress. (H2) Higher psychological needs satisfaction is positively associated with adaptive humor styles (affiliative and self-enhancing) and negatively associated with maladaptive humor styles (self-defeating and aggressive). (H3) Adaptive humor styles are associated with lower levels of distress, while maladaptive humor styles are associated with higher levels of distress. (H4) Humor styles will statistically account for part of the association between psychological needs satisfaction and psychological distress. (H5) Sex will moderate all associations, including those between psychological needs satisfaction and psychological distress (c′-path), psychological needs satisfaction and humor (a-path), and those between humor styles and psychological distress (b-path). However, the strength of these relationships will differ between males and females.

## 2 Materials and methods

### 2.1 Participants and procedures

From March 2016 to March 2018, students aged 18 to 30 without a history of psychiatric disorders from the Medical University and the Leopold-Franzens University of Innsbruck, Austria were invited to participate in the study. Of those invited, a total of 226 students from different faculties (mainly medicine, psychology, and social sciences) completed a battery of psychological assessments in an online survey. The students were of various nationalities, with the majority being Austrian (54.4%), followed by German (32.3%) and Italian (10%). All procedures contributing to this work complied with the standards of the local ethics committee and were conducted according to the standards of Good Clinical Practice (GCP) on human experimentation, as well as the Helsinki Declaration of 1975, revised in 2008. The study was approved by the ethics committee of the Medical University Innsbruck and all participants provided written informed consent.

### 2.2 Measures

Self-determination was measured using the 18-item Balanced Measure of Psychological Needs (BMPN) (Sheldon and Hilpert, 2012), which covers the domains of autonomy, competence, and relatedness (Deci and Ryan, 2000; Neubauer and Voss, 2016). Rated on a 5-point Likert-type scale (1 = strongly disagree, 5 = strongly agree), these dimensions reflect volition, self-efficacy, and social connectedness and should not be combined into a single general needs factor (Ryan and Deci, 2000; Sheldon and Gunz, 2009; Neubauer and Voss, 2016). The BMPN does not ask about the specific means of meeting these needs. Reliability analyses of the six three-item BMPN subscales after reverse-scoring the negatively worded items resulted in the following coefficients: *α* = 0.78 for BMPN autonomy, *α* = 0.79 for BMPN competence, and *α* = 0.78 for BMPN relatedness (Sheldon and Hilpert, 2012).

Humor was assessed using the Humor Styles Questionnaire (HSQ) (Martin et al., 2003), which distinguishes between affiliative and self-enhancing (positive) humor, as well as aggressive and self-defeating (negative) humor. Affiliative humor is characterized as empathic and fosters interpersonal relationships. Self-enhancing humor aids in coping and taking a perspective-based approach to life. In contrast, aggressive humor disregards others, and self-defeating humor, mainly used as a defensive mechanism, involves self-denigration (Martin et al., 2003). Each humor style is rated using eight items on a 7-point Likert-type scale (1 = strongly disagree, 7 = strongly agree), and higher scores indicate greater usage. The HSQ has been demonstrated to exhibit adequate internal consistency, as evidenced by Cronbach alpha coefficients ranging from 0.77 to 0.81. The internal consistency of the HSQ domains was acceptable to good, with the following values: *α* = 0.80 for affiliative humor, *α* = 0.81 for self-enhancing humor, *α* = 0.77 for aggressive humor, and *α* = 0.80 for self-defeating humor (Martin et al., 2003).

Psychological distress was assessed using the 53-item German version of the Brief Symptom Inventory (BSI) (Derogatis, 1993; Geisheim et al., 2002). Responses were given on a 5-point Likert scale (0 = no distress, 4 = extreme distress). This scale evaluates nine mental health domains (anger-hostility, anxiety, depression, paranoid ideation, phobic anxiety, psychoticism, somatization, interpersonal sensitivity, and obsessive-compulsiveness) and provides three global distress indices. The Global Severity Index (GSI), which was used in this study, is the most sensitive index, integrating symptom count and distress intensity. Based on sex-differentiated student norms, a T-score ≥ 63 indicates clinically relevant distress (Franke, 2000). The scale has demonstrated acceptable to good internal consistency for all subscales, with Cronbach’s *α* ranging from 0.70 to 0.89 and with excellent external consistency the GSI score *α* = 0.96 (Geisheim et al., 2002).

### 2.3 Statistics

Statistical analysis was conducted using IBM SPSS Statistics, version 29.0.0 statistical software (IBM Corp, 2022). For the BSI, age- and sex-specific normative *T-values* were used to ensure comparability across participants. A series of *t-tests* for independent samples was performed to compare male and female participants regarding age, psychological needs satisfaction, psychological distress, and HSQ subscale scores. When relevant, the mean difference (MD) between the two groups was reported along with 95% confidence intervals (CIs), associated *p-values*, and Cohen’s *d* as a measure of effect size. We corrected for multiple testing by providing Benjamini-Hochberg corrected *p-values*. Chi^2^ tests were used to calculate potential sex differences in categorical sociodemographic data.

Effect sizes were interpreted according to Cohen’s (Cohen, 2013) guidelines as follows: small (*d* ≥ 0.2), medium (*d* ≥ 0.5), and large (*d* ≥ 0.8). Pearson’s product–moment correlation coefficient (*r*) was used to evaluate the strength and direction of the linear relationship between variables. Fisher’s *r-*to-*z* transformation was applied to compare correlation coefficients between male and female participants.

The analysis primarily focused on mediation and moderation modeling. The PROCESS macro (Hayes, 2013) was used to estimate model parameters and assess indirect and interaction effects. First, a simple mediation analysis (model 4) was conducted to determine if different humor styles (mediator, *M*) statistically accounted for part of the association between the satisfaction of psychological needs (independent variable, *X*) and psychological distress (dependent variable, *Y*). Since the BMPN and the HSQ assess different domains of psychological needs and humor styles, global individual factors or total scores could not be used for an overall assessment.

To account for the potential moderating effect of sex, a moderated mediation analysis was conducted. As proposed by Hayes (2013), two distinct models were tested. In model 7, psychological needs satisfaction (*X*) was the independent variable, perception of distress (*Y*) the dependent variable, humor (*M*) the mediator, and sex (*W*) the moderator of the relationship between psychological needs satisfaction and humor. In model 15, sex was entered as a moderator between psychological needs satisfaction (*X*) and psychological distress (*Y*), as well as between humor (*M*) and psychological distress (*Y*). In addition, the effect of age was tested as a covariate in model 15. The estimation of path coefficients, direct, indirect, and total effects was facilitated by ordinary least square regressions. Bias-corrected, bootstrapped (10,000 resamples) 95% confidence intervals based on heteroscedasticity-consistent standard errors (HC3) (Davidson and MacKinnon, 1993) were used to test the indirect effects. The effects were considered statistically significant when the confidence interval did not include zero (Hayes, 2013). Prior to the mediation analyses, all continuous variables were standardized using *z*-scores.

## 3 Results

### 3.1 Descriptive statistics

The final sample consisted of 226 university students, of whom 152 were female (67.3%) and 74 were male. Mean age was 22.3 years (*SD* = 2.59), and males were significantly older than females (*MD* = 1.31, *p =* 0.001). Most participants were enrolled in medical studies (67.6% of males vs. 53.3% of females) and lived in shared accommodations (58.1% of males vs. 57. 9% of females). A chi-square test did not identify any other significant sex differences with respect to the assessed sociodemographic variables, including citizenship, partnership status, and employment status. Detailed sociodemographic characteristics are provided in Table 1.

**Table 1:** Sample characteristics.

Variable		Male *N* = 74		Female *N* = 152		χ^2^*(df)*	*p-value*	φ
		*N* or *Mean*	% or *SD*	*N* or *Mean*	% or *SD*			
Age	Years	23.18	2.59	21.86	2.38		**0.001**	
Citizenship	Austria	39/74	52.70	84/152	55.26			
	Germany	26/74	35.14	47/152	30.92	1.12 (3)	0.77	0.07
	Italy	6/74	8.11	17/152	11.18			
	Others	3/74	4.05	4/152	2.63			
Marital status	Stable partnership	37/74	50.00	73/152	48.00	0.08 (1)	0.78	0.02
	Single	37/74	50.00	79/152	52.00			
Living Condition	Alone	15/74	20.27	19/152	12.50			
	With parents	6/74	8.11	28/152	18.42			
	With partner/ own family	8/74	10.81	12/152	7.89	6.05 (4)	0.19	0.16
	Flat-sharing	43/74	58.11	88/152	57.89			
	Others	2/74	2.70	5/152	3.29			
Employment status	Student with or without part-time job	72/74	97.30	150/152	98.68			
	Mainly employed with part-time studies	2/74	2.70	2/152	1.32	0.55 (1)	0.46	0.05
Study program	Psychology	7/74	9.46	19/152	12.50			
	Medicine	50/74	67.57	81/152	53.29			
	Social work	1/74	1.35	18/152	11.84	32.09 (25)	0.16	0.38
	Education and educational sciences	2/74	2.70	7/152	4.61			
	Others	14/74	18.92	27/152	17.76			

Bold values indicate statistically significant correlations or coefficients (*p* < .05), including corrected *p*-values where applicable.

According to mean GSI T-scores, psychological distress showed no significant sex differences. However, compared to females, males scored significantly higher in the autonomy domain of the BMPN and reported affiliative and aggressive humor styles more frequently. No sex differences were observed in the other BMPN domains or in self-enhancing and self-defeating humor styles (see Table 2).

**Table 2:** Humor styles, psychological needs satisfaction, and psychological distress in male and female study participants.

Scale	Sex	Mean	SD	*p-value* / *p_BH_-value*	*t*	*df*	*d*
BMPN relatedness	Male	23.32	2.51	0.78/0.78	1.77	219	0.25
	Female	22.66	2.68				
BMPN competence	Male	23.29	4.44	0.22/0.34	1.22	219	0.17
	Female	22.54	4.29				
BMPN autonomy	Male	24.34	3.70	**0.01/0.03**	2.59	219	0.37
	Female	22.82	4.30				
HSQ affiliative	Male	48.36	6.28	**0.02/0.048**	2.28	219	0.33
	Female	46.21	6.79				
HSQ self-enhancing	Male	39.12	8.80	0.59/0.793	0.53	219	0.76
	Female	38.46	8.64				
HSQ aggressive	Male	30.23	7.94	**0.001/0.004**	3.38	219	0.48
	Female	26.50	7.66				
HSQ self-defeating	Male	24.78	8.35	0.91/0.91	0.12	219	0.17
	Female	24.65	8.02				
BSI GSI T-score	Male	49.77	8.02	0.44	0.78	219	0.11
	Female	50.73	8.88				

*N* = 221 (males *N* = 74, females *N* = 147).

BMPN, basic measure of psychological needs; HSQ, humor styles questionnaire; BSI GSI, brief symptom inventory global severity index; *d*, Cohen’s effect size; *p__BH__-value – p-value* with Benjamini-Hochberg correction. Bold values indicate statistically significant correlations or coefficients (*p* < .05), including corrected *p*-values where applicable.

### 3.2 Correlations between psychological needs satisfaction, humor, and psychological distress

Considering the entire sample, weak positive correlations were found between an affiliative humor style and the BMPN domains of relatedness (*r* = 0.16, *p =* 0.006) and autonomy (*r =* 0.18, *p =* 0.007). A self-enhancing humor style, on the other hand, correlated weakly positively with all BMPN domains, i.e., with relatedness (*r =* 0.32, *p <* 0.001), competence (*r =* 0.29, *p <* 0.001), and autonomy (*r =* 0.28, *p <* 0.001). There were weak negative correlations between a self-defeating humor style and all BMPN domains (relatedness: *r =* −0.21, *p =* 0.002; competence: *r =* −0.27, *p <* 0.001; autonomy: *r =* −0.24, *p <* 0.001). However, no correlation was found between an aggressive humor style and psychological needs satisfaction.

Psychological distress (BSI GSI) was moderately positively correlated with a self-defeating humor style (*r =* 0.33, *p <* 0.001) and moderately negatively correlated with a self-enhancing humor style (*r =* −0.40, *p <* 0.001). In addition, moderate to strong negative correlations were detected between psychological distress and all BMPN domains (relatedness: *r =* −0.51, *p <* 0.001; competence: *r =* −0.47, *p <* 0.001; autonomy: *r =* −0.55, *p <* 0.001). A detailed presentation of these correlations is provided in Table 3.

**Table 3:** Pearson correlations between humor styles, psychological needs satisfaction, and psychological distress.

Whole sample *N* = 221	Correlation(*p- / pBH-value*)	BMPN-Relatedness	BMPN-Competence	BMPN-Autonomy	HSQ - Affiliative Humor	HSQ - Self-Enhancing Humor	HSQ - Aggressive Humor	HSQ - Self-Defeating Humor
BMPN-Competence	Pearson	0.43**						
	*p- / p_BH_-value*	<0.001/ <0.001						
BMPN-Autonomy	Pearson	0.42**	0.41**					
	*p- / p_BH_-value*	<0.001/ <0.001	<0.001/ <0.001					
HSQ - Affiliative Humor	Pearson	0.19**	0.09	0.18**				
	*p- / p_BH_-value*	0.01/0.01	0.20/0.20	0.01/0.01				
HSQ - Self-Enhancing Humor	Pearson	0.32**	0.29**	0.28**	0.42**			
	*p- / p_BH_-value*	<0.001/ <0.001	<0.001/ <0.001	<0.001/ <0.001	<0.001/ <0.001			
HSQ - Aggressive Humor	Pearson	−0.043	−0.10	−0.12	0.11	−0.024		
	*p- / p_BH_-value*	0.53/0.53	0.15/0.19	0.08/0.08	0.10/0.10	0.73/0.73		
HSQ - Self-Defeating Humor	Pearson	−0.21**	−0.27**	−0.24**	0.02**	0.04**	0.21**	
	*p- / p_BH_-value*	<0.001/ <0.001	<0.001/ <0.001	<0.001/ <0.001	<0.001/ <0.001	<0.001/ <0.001	<0.001/ <0.001	
BSI Global Severity Index	Pearson	−0.51**	−0.47**	−0.55**	−0.10	−0.40**	0.09	0.33**
	*p- / p_BH_-value*	<0.001/ <0.001	<0.001/ <0.001	<0.001/ <0.001	0.16	<0.001/ <0.001	0.176	<0.001/ <0.001

**The correlation is significant at the level of 0.01 (2-tailed). Bold values indicate statistically significant correlations or coefficients (*p* < .05), including corrected *p*-values where applicable. *p_BH_-value* – Benjamini & Hochberg Correction (False Discovery Rate).

When comparing sexes, in both females and males, a self-defeating humor style was found to correlate significantly negatively with all BMPN domains, which, in turn, correlated significantly positively with each other. Additionally, psychological distress was found to correlate significantly negatively with all BMPN domains and significantly positively with a self-defeating humor style in both sexes. Further correlations broken down by sex are shown in Table 4. Age did not show a significant correlation with any of the examined psychological variables in either sex.

**Table 4:** Pearson correlations between humor styles, psychological needs satisfaction, and perceived distress among male and female participants.

Males *N* = 74		1	2	3	4	5	6	7
2	Pearson	0.34**						
	*p- / p_BH_-value*	0.003/0.003						
3	Pearson	0.42**	0.33**					
	*p- / p_BH_-value*	<0.001/ <0.001	0.015					
4	Pearson	0.14	−0.12	0.20				
	*p- / p_BH_-value*	0.24/0.32	0.31/0.31	0.09/0.11				
5	Pearson	0.30*	0.23	0.18	0.37**			
	*p- / p_BH_-value*	0.01/0.02	0.05/0.11	0.12/0.12	0.001/0.003			
6	Pearson	0.02	−0.17	−0.21	0.09	−0.05		
	*p- / p_BH_-value*	0.88/0.88	0.15/0.19	0.08/0.11	0.44/0.66	0.65/0.96		
7	Pearson	−0.31**	−0.35**	−0.47**	0.01	0.03	0.35**	
	*p- / p_BH_-value*	0.01/0.02	0.002/0.01	<0.001/ <0.001	0.96/0.96	0.82/0.96	0.002/0.01	
8	Pearson	−0.58**	−0.50**	−0.49**	−0.06	−0.23	0.09	0.46**
	*p- / p_BH_-value*	<0.001/ <0.001	<0.001/ <0.001	<0.001/ <0.001	0.60/0.60	0.05/0.11	0.43/0.57	<0.001/ <0.001

Females *N* = 147		1	2	3	4	5	6	7
2	Pearson	0.47**						
	*p- / p_BH_-value*	<0.001/ <0.001						
3	Pearson	0.40**	0.44**					
	*p- / p_BH_-value*	<0.001/ <0.001	<0.001/ <0.001					
4	Pearson	0.18*	0.17*	0.14				
	*p- / p_BH_-value*	0.03/0.05	0.04/0.06	0.09/0.09				
5	Pearson	0.32**	0.33**	0.32**	0.45**			
	*p- / p_BH_-value*	<0.001/ <0.001	<0.001/ <0.001	<0.001/ <0.001	<0.001/ <0.001			
6	Pearson	−0.12	−0.09	−0.15	0.07	−0.02		
	*p- / p_BH_-value*	0.17/0.17	0.26/0.26	0.08/0.09	0.38/0.58	0.80/0.80		
7	Pearson	−0.17*	−0.22**	−0.15	0.02	0.05	0.14	
	*p- / p_BH_-value*	0.04/0.06	0.01/0.01	0.07/0.09	0.79/0.79	0.58/0.80	0.09/0.26	
8	Pearson	−0.47**	−0.45**	−0.55**	−0.08	−0.47**	0.15	0.28**
	*p- / p_BH_-value*	<0.001/ <0.001	<0.001/ <0.001	<0.001/ <0.001	0.39/0.36	<0.001/ <0.001	0.07/0.09	0.001/0.002

**The correlation is significant at the level of 0.01 (2-tailed).

*The correlation is significant at the level of 0.05 (2-tailed).

Numbering: 1. BMPN Relatedness 2. BMPN Competence 3. BMPN Autonomy 4. HSQ – Affiliative Humor 5. HSQ - Self-Enhancing Humor 6. HSQ – Aggressive Humor 7. HSQ - Self-Defeating Humor 8. BSI GSI.

BMPN, basic measure of psychological needs; HSQ, humor styles questionnaire; BSI GSI, brief symptom inventory global severity index. *p_BH_-value* – Benjamini & Hochberg Correction (False Discovery Rate).Bold values indicate statistically significant correlations or coefficients (*p* < .05), including corrected *p*-values where applicable.

Significant sex differences emerged in terms of two specific correlations. First, a positive correlation was seen between the BMPN domain of competence and an affiliative humor style in females, but not in males (*z* = −2.00, *p* = 0.046). Conversely, the BMPN domain of autonomy significantly negatively correlated with a self-defeating humor style in males only (*z* = −2.48, *p* = 0.013). A significantly stronger correlation was found between the BMPN domain of autonomy and an affiliative humor style in males (*z* = −2.38, *p* = 0.017), though it was not meaningful for each sex separately. No further statistically significant sex differences were observed (see Table 5).

**Table 5:** Pearson correlations between humor styles, psychological needs satisfaction, and psychological distress: comparison of sexes.

Fisher’s r-to-z transformation Males *N* = 74 vs. Females *N* = 147		BMPN-Relatedness	BMPN-Competence	BMPN-Autonomy	HSQ - Affiliative Humor	HSQ - Self-Enhancing Humor	HSQ - Aggressive Humor	HSQ - Self-Defeating Humor
BMPN-competence	*z*	−0.89						
	*p*-value	0.37						
BMPN-autonomy	*z*	0.19	−0.95					
	*p*-value	0.85	0.34					
HSQ - affiliative humor	*z*	−0.33	−2*	−2.38*				
	*p*-value	0.74	0.05	0.017				
HSQ - self-enhancing humor	*z*	−0.19	−0.76	−0.98	−0.66			
	*p*-value	0.85	0.45	0.33	0.51			
HSQ - aggressive humor	*z*	0.91	−0.54	−0.43	0.14	−0.21		
	*p*-value	0.36	0.59	0.67	0.89	0.83		
HSQ - self-defeating humor	*z*	−1.03	−0.94	−2.48*	0.19	−0.14	1.58	
	*p*-value	0.30	0.35	0.013	0.85	0.89	0.11	
BSI global severity index	*z*	−0.98	−0.39	0.56	0.09	1.92	−0.40	1.42
	*p*-value	0.33	0.70	0.58	0.93	0.06	0.69	0.16

*The correlation is significant at the level of 0.05 (2-tailed).Bold values indicate statistically significant correlations or coefficients (*p* < .05), including corrected *p*-values where applicable.

### 3.3 Humor as a mediator of the relation between psychological needs satisfaction and psychological distress

**Figure 1:**
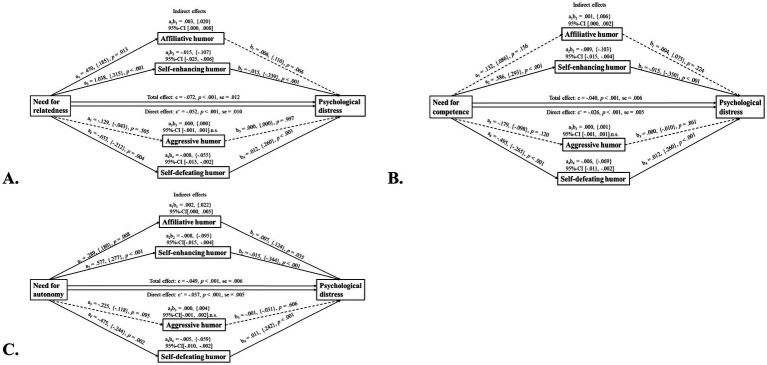
Humor styles as mediators of psychological needs satisfaction **(A)** Relatedness, **(B)** Competence, **(C)** Autonomy and psychological distress in emerging adults. n.s., not significant; se, standard error. *N* = 221; male subjects: *N* = 74, female subjects: *N* = 147. Values in curved brackets represent completely standardized coefficients (b) for metric variables. Solid lines indicate statistically significant effects, dashed lines indicate non-significant effects.

Figure 1 illustrates the results of the mediation analyses with psychological needs satisfaction (A: relatedness, B: competence, C: autonomy) as the independent variable (*X*), psychological distress as the dependent variable (*Y*), and humor as the mediator (*M*). The results show that the influence of the satisfaction of each psychological need on perceived distress was only partially attributable (around 30% of the total effect in relatedness, competence, and autonomy) to the mediating effects of the use of affiliative (*a_1_ × b_1_*: less than 5% of the total effect), self-enhancing (*a_2_ × b_2_*: 20.83, 22.5, and 16.33% of the total effect in relatedness, competence, and autonomy, respectively), and self-defeating (*a_4_ × b_4_*: 11.11, 15, and 10.2% of the total effect in relatedness, competence, and autonomy) humor styles. No statistically significant results were obtained for the indirect effect of aggressive humor (*a_3_ × b_3_*). However, the direct effect remained significant (relatedness: *c’* = −0.05, *p* < 0.001; competence: *c’* = −0.03, *p* < 0.001; autonomy: *c’* = −0.04, *p* < 0.001). These effects explained 72, 65, and 75.5%, respectively, of the total variance in perceived distress.

**Figure 2:**
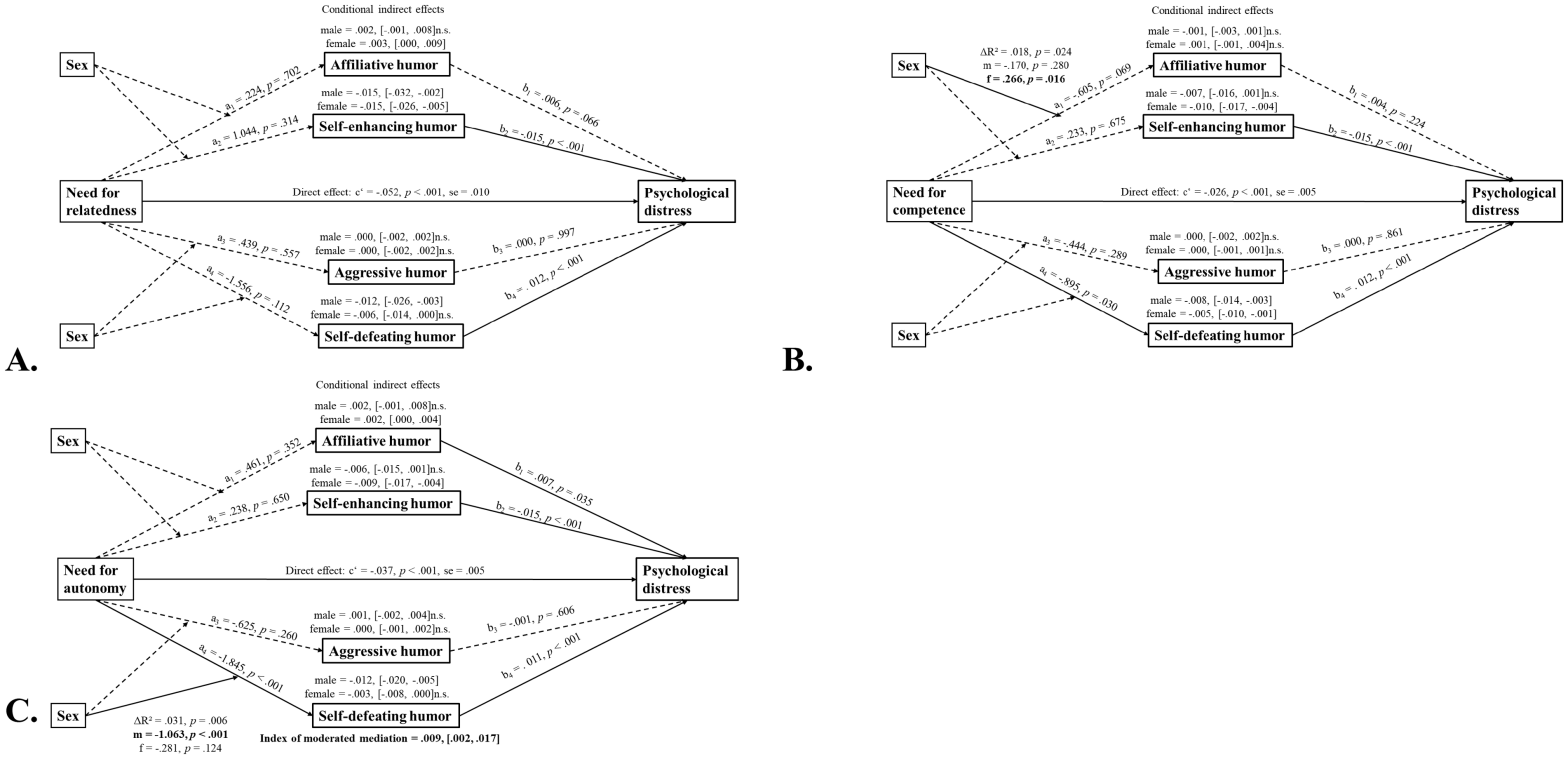
Conditional indirect effects of psychological needs satisfaction on psychological distress via different humor styles in the two sexes (Hayes’s model 7). n.s., not significant; se, standard error. *N* = 221; male subjects: *N* = 74, female subjects: *N* = 147. Solid lines indicate statistically significant effects, dashed lines indicate non-significant effects.

To test whether sex moderated the relationships between psychological needs satisfaction and humor, humor and psychological distress, and psychological needs satisfaction and psychological distress, two different models of those proposed by Hayes (2013) were tested. BMPN domains were again entered as the independent variable, psychological distress as the dependent variable, and humor as the mediator between these variables. In model 7, sex was entered as the moderator between psychological needs satisfaction and humor (Figure 2). A significant moderated mediation effect was found only in the autonomy-self-defeating humor style – psychological distress pathway. The interaction term between autonomy and sex was significant (unstandardized interaction coefficient *t =* −19.60, *SE* = 0.007, *t* = −2.89, *p* = 0.004). The index of moderated mediation was 0.009 (95% CI: 0.002, 0.017), indicating statistically significant moderation by sex. Conditional indirect effects revealed a stronger negative effect in males (effect = −0.012, 95% CI: −0.020, −0.005). This suggests that the mediation of a self-defeating humor style on the effect of autonomy on psychological distress is stronger in males than in females. The relationship between competence and an affiliative humor style was significantly stronger in females, however, the indirect effect of an affiliative humor style on psychological distress was not significant.

**Figure 3:**
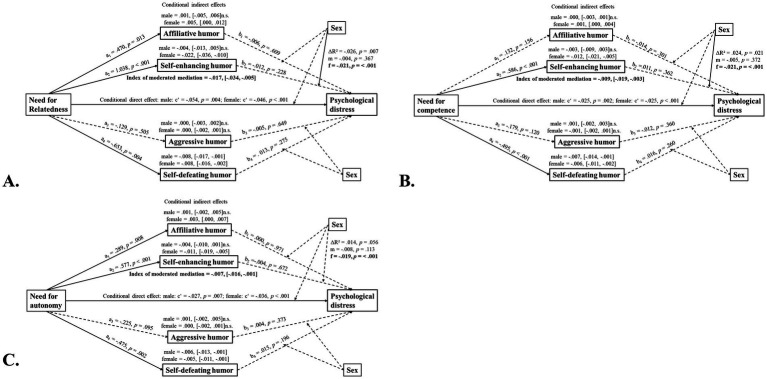
Conditional indirect effects of psychological needs satisfaction and psychological distress via different humor styles in the two sexes (Hayes’s model 15). n.s., not significant; se, standard error. *N* = 221; male subjects: *N* = 74, female subjects: *N* = 147. Solid lines indicate statistically significant effects, dashed lines indicate non-significant effects.

In model 15, sex was tested as a moderator in the second stage of the mediation model (i.e., from humor style to psychological distress) and in the direct path between psychological needs satisfaction and psychological distress (Figure 3).

The influence of sex was evident in the relationship between a self-enhancing humor style and psychological distress, with females scoring significantly higher than males. The indices of moderated mediation for all combinations of psychological needs satisfaction and humor styles included zero in their confidence intervals. This indicates that the indirect effects of self-enhancing humor on the relationship between psychological needs satisfaction and distress were sex-dependent. Adding age as a covariate did not significantly change the results; no coefficients shifted by more than 0.01.

In summary, these findings suggest that, while sex moderates the link between autonomy and self-defeating humor (model 7), the associations between psychological needs satisfaction, humor styles, and psychological distress differ partially between sexes (model 15). More specifically, this study revealed significant sex-moderated mediation effects in the relationship between psychological needs satisfaction and psychological distress via self-enhancing humor. The indices of moderated mediation were 0.017 for the relationship between relatedness and psychological distress, 0.009 for the relationship between competence and psychological distress, and 0.007 for the relationship between autonomy and psychological distress.

## 4 Discussion

This study examined the relationship between psychological needs satisfaction, humor styles, and psychological distress during emerging adulthood. The study focused specifically on humor as a potential explanatory mechanism and on sex differences in these pathways. Based on SDT (Ryan and Deci, 2000; Ryan and Deci, 2017), the findings provide converging evidence that satisfaction of the three basic psychological needs - autonomy, competence, and relatedness - is strongly associated with lower psychological distress in young adults (confirming H1). Among these needs, relatedness was found to be the strongest correlate of distress. This finding highlights the central role of social connectedness during a developmental period marked by identity exploration and relational instability (Arnett, 2000; Arnett, 2007; Yalcin-Siedentopf et al., 2021).

Consistent with SDT and in line with H2, higher psychological needs satisfaction was associated with greater use of adaptive humor styles and less use of maladaptive humor styles. Specifically, self-enhancing humor was positively related to autonomy, competence, and relatedness, whereas self-defeating humor was negatively related to all three needs. These results support the idea that humor styles are stable ways of regulate emotions and coping with interpersonal situations that fit within individuals’ broader motivational context (Martin, 2007; Samson and Gross, 2012).

From a motivational perspective, satisfying basic psychological needs fosters psychological resources such as self-acceptance, adaptive emotion regulation, and flexible coping. These resources are essential for psychological adjustment and well-being (Vansteenkiste and Ryan, 2013; Ryan and Deci, 2017). The present results suggest that these resources may be reflected in habitual humor use, with self-enhancing humor representing an adaptive way to maintain positive feelings under stress and self-defeating humor reflecting compromised self-regulation and heightened vulnerability to negative self-evaluation. Affiliative humor in present findings was positively associated with relatedness and autonomy, but not with competence or psychological distress which is why H3 could be confirmed only partially. One possible explanation for this is that affiliative humor primarily serves an interpersonal function, aiming to maintain social harmony and strengthen relationships rather than regulate individual emotional states (Martin et al., 2003; Martin, 2007). In turn, relatedness reflects the need for social connection and belonging, which is consistent with its strong association with affiliative humor (Martin et al., 2003; Martin, 2007). The weaker association with autonomy may reflect the role of self-expression and voluntary social interaction in humor-based communication (Ryan and Deci, 2000; Ryan and Deci, 2017). In contrast, competence refers to feelings of effectiveness and mastery, which may be less directly connected to humor use in social contexts. Similarly, affiliative humor may contribute more to interpersonal adjustment than to intrapersonal emotional regulation, which could explain its limited association with psychological distress in the present study. Previous research has likewise reported relatively modest or inconsistent relationships between affiliative humor and mental health outcomes, suggesting that this humor style may function primarily as a social bonding strategy rather than a direct buffer against psychological distress (Cann et al., 2008; Martin and Ford, 2018).

Contrary to expectations, aggressive humor did not show significant associations with either psychological need satisfaction or psychological distress. One possible explanation is that aggressive humor is highly context-dependent and serves different social functions depending on interpersonal dynamics and situational factors. Unlike self-enhancing or self-defeating humor, which primarily reflect intrapersonal emotion-regulation strategies, aggressive humor is typically directed toward others and can be interpreted differently based on the context of the relationship (Cann et al., 2008; Martin and Ford, 2018). For instance, teasing and sarcasm among close friends may be perceived as playful or bonding; however, similar behavior in less established relationships may be perceived as hostile or exclusionary (Dynel, 2009). Furthermore, the social consequences of disparaging humor depend strongly on group norms and the perceived acceptability of the target (Ford et al., 2001; Ford and Ferguson, 2004). This contextual variability may weaken the overall association between aggressive humor and psychological outcomes in cross-sectional, self-report studies. This interpretation aligns with previous findings indicating that aggressive humor is less consistently associated with psychological well-being than other humor styles (Kuiper et al., 2004).

Important factor that may influence the associations observed in the present study is cultural context. The sample was drawn from Austria, a country generally characterized as relatively individualistic in cross-cultural research (Hofstede, 2001). Cultural norms may shape both the expression and interpretation of humor styles as well as the salience of psychological needs. For example, affiliative and self-enhancing humor may be particularly valued in individualistic contexts that emphasize personal expression and positive self-regulation, whereas collectivistic cultures may place greater emphasis on maintaining social harmony and avoiding behaviors that could disrupt group cohesion (Hofstede, 2001; Yue et al., 2016; Martin and Ford, 2018). Similarly, aggressive humor may be perceived differently depending on cultural norms regarding hierarchy, interpersonal boundaries, and acceptable forms of teasing. These cultural variations may influence how humor styles relate to psychological needs and psychological distress. Future research should therefore examine these associations across diverse cultural contexts to better understand the extent to which the observed patterns generalize beyond individualistic societies.

Building on earlier research, our findings suggest that different types of humor play a role in the relationship between meeting psychological needs and experiencing psychological distress. Consistent with H4, self-enhancing and self-defeating humor styles partially accounted for part of the association between needs satisfaction and distress across all three psychological needs. These results align with existing evidence indicating that humor may be a psychological process linking cognitive and motivational factors to emotional outcomes (Dozois et al., 2009).

Importantly, given the cross-sectional nature of the data, this mediation should be interpreted statistically rather than causally. The results suggest that individuals who are more satisfied with their lives tend to exhibit humor styles associated with lower distress, while those who are less satisfied tend to exhibit humor styles associated with greater distress. Thus, humor styles may be one of several behavioral and emotional pathways through which motivational states are associated with psychological well-being during emerging adulthood. This interpretation aligns with SDT emphasis on indirect pathways linking motivation to mental health via self-regulatory processes (Vansteenkiste and Ryan, 2013).

Consistent with H5, several associations differed by sex, underscoring the importance of considering sex-related processes in motivational and coping research. Notably, the indirect association between autonomy satisfaction and psychological distress via self-defeating humor was stronger among males than females. This suggests that unmet autonomy needs may be particularly salient for men, potentially leading them to use maladaptive coping strategies, such as self-defeating humor. Previous studies have also identified autonomy as a significant predictor of emotional well-being in men (Saroglou and Scariot, 2002) and associated autonomy frustration with maladaptive coping behaviors (Frewen et al., 2008).

In contrast, the indirect associations between psychological needs satisfaction - particularly relatedness and competence - and distress via self-enhancing humor were stronger in females. This suggests that self-enhancing humor may play a more significant protective role for women. This finding aligns with research indicating that women tend to employ emotion-focused coping strategies and positive reappraisal more frequently (Matud, 2004; Nolen-Hoeksema, 2012). Although affiliative humor was more strongly associated with competence in females, it did not result in sex-specific differences in distress. This aligns with previous findings that affiliative humor is only weakly related to mental health outcomes (Martin et al., 2003).

Taken together, these findings underscore the importance of psychological needs satisfaction for mental health during emerging adulthood. They also suggest that humor styles may be meaningful, albeit partial, pathways linking motivation and distress. The results also underscore the importance of considering sex-specific patterns when examining coping processes. Preventive and intervention approaches may benefit from fostering autonomy-supportive environments for men to reduce their reliance on self-defeating humor. Promoting self-enhancing humor and needs satisfaction, particularly relatedness and competence, may be especially beneficial for women.

There are several limitations that should be considered. First, the cross-sectional design restricts the ability to make causal inferences. While the mediation analyses offer insight into potential pathways linking motivational factors and coping tendencies to psychological outcomes, they cannot establish the temporal ordering of these variables. Longitudinal studies would be valuable for examining the developmental dynamics of these relationships during emerging adulthood. These studies could clarify whether the satisfaction of psychological needs prospectively predicts changes in humor styles, and whether these changes subsequently influence trajectories of psychological distress. Second, the extended data collection period could introduce potential cohort and history effects. However, since the constructs assessed in this study reflect stable psychological tendencies rather than short-term states, it is unlikely that temporal fluctuations substantially affected the observed associations. Nevertheless, future research would benefit from shorter data collection periods or controlling for time-related variables. Longitudinal designs and repeated cross-sectional studies may help identify potential temporal or contextual influences on these relationships. Focusing exclusively on university students, most of whom were enrolled in health-related disciplines and were recruited from a limited geographic region, restricts the generalizability of the results to other emerging adult populations. Although studying emerging adulthood in student populations is common due to the developmental relevance of this life stage (Arnett, 2000), this approach excludes individuals who are not pursuing higher education and those in different socioeconomic contexts. University students may face unique academic and social challenges that affect their psychological well-being and coping mechanisms, including the use of humor. Therefore, examining larger and more heterogeneous samples would allow for a more precise analysis of the potential impact of context on the relationships between motivational factors, humor styles, and psychological distress. Another limitation is the reliance on self-report measures to assess humor styles, psychological needs satisfaction, and psychological distress. While the instruments used in this study are well-established and widely validated, self-report data can be affected by response biases, such as social desirability bias, shared method variance, and discrepancies between individuals’ self-perceptions and how their behaviors are perceived by others. In particular, humor styles are socially expressed behaviors that may not always be accurately captured through self-assessment alone (Greengross and Miller, 2011). Future research could incorporate observer-rated measures, peer reports, behavioral assessments of humor use in social interactions or control for personality traits such as self-esteem and narcissism, as suggested by Ruch and Heintz (2013). Finally, sex was operationalized as a binary variable. Although this approach enabled the examination of sex differences in the present study, it does not fully capture the complexity of gender-related processes that may influence coping strategies, humor usage, and psychological well-being. Contemporary psychological research increasingly emphasizes distinguishing biological sex from gender identity, gender roles, and gender-related socialization processes (APA, 2015; Hyde et al., 2019). Future studies should incorporate more nuanced measures of gender identity and gender roles to better understand how gender-related experiences influence relationships between psychological needs satisfaction, humor styles, and psychological distress. Including such measures may help clarify whether the observed differences reflect biological factors, gender-related socialization patterns, or broader sociocultural influences on coping and emotional regulation.

Despite these limitations, the present study contributes to a more nuanced understanding of the interrelationship between motivational factors, coping styles, and psychological distress are interrelated in emerging adulthood. Integrating SDT with the humor styles framework highlights humor as a psychologically meaningful correlate of needs satisfaction and distress. This underscores the importance of sex-sensitive approaches in research and prevention efforts targeting the mental health of young adults.

## Data Availability

The raw data supporting the conclusions of this article will be made available by the authors, without undue reservation.
